# Synthesis and Characterization of Na-P1 (GIS) Zeolite Using Rice Husk

**DOI:** 10.3390/molecules29235596

**Published:** 2024-11-26

**Authors:** Daniela Novembre, Domingo Gimeno, Lucia Marinangeli, Anna Chiara Tangari, Gianluigi Rosatelli, Michele Ciulla, Pietro di Profio

**Affiliations:** 1 Dipartimento di Scienze, Università degli Studi “G. d’Annunzio”, Via dei Vestini 30, 66013 Chieti, Italy; lucia.marinangelii@unich.it (L.M.); a.tangari@unich.it (A.C.T.); gianluigi.rosatelli@unich.it (G.R.); 2 Department Mineralogia, Petrologia i Geologia Aplicada, Universitat de Barcelona, 08028 Barcelona, Spain; d.gimeno.torrente@gmail.com; 3 Department of Pharmacy, University of Chieti-Pescara “G. d’Annunzio”, Via dei Vestini, 66100 Chieti, Italypietro.diprofio@unich.it (P.d.P.)

**Keywords:** rice husk, synthesis, zeolite Na-P1

## Abstract

This work deals with the synthesis of Na-P1 (GIS) zeolite using rice husk as the starting material, instead of the more expensive chemicals currently used in the industry (i.e., Na aluminates and Na silicates). Rice husk is calcined at the temperature of 550 °C to obtain rice husk ash. Na-P1 is synthesized starting from rice husk ash, NaOH, and NaAlO_2_ by a protocol involving the mixing of a seed gel and a feedstock gel. Two synthesis runs are carried out at ambient pressure at the temperature of 110 °C by fixing the SiO_2_/Al_2_O_3_ ratio at 3.5 and 5.3, respectively. The synthesized products have been identified as well as the experiments developed by X-ray diffraction and scanning electron microscopy. Then, the most successful synthesized powders were also characterized by infrared spectroscopy, Raman spectroscopy, specific surface area (BET), and differential thermal analysis. The cell parameters are calculated using the Rietveld method. The combined Rietveld and reference intensity ratio methods allows us to exclude the presence of impurities and residual amorphous phase in the conducted experiments. Testing rice husk as a source of amorphous silica in the synthesis of Na-P1 represents both economic and environmental advantages. The high yields and the results of the experiment open the way for the transfer to an industrial production scale.

## 1. Introduction

Although zeolites constitute a group of natural tectosilicates that have been known since the mid-18th century, and some of their properties began to be known a century later, it was only in 1948 when Union Carbide began to synthesize them with industrial objectives that awareness of them developed exponentially. Today, we know about 100 naturally occurring minerals with synthetic analogs now reaching more than 250 species. As is well knowledge of these minerals, the crystallochemistry of these types of tectosilicates is characterized by three-dimensional frameworks of Al/Si tetrahedra linked by sharing their oxygen atoms to form channels containing water and exchangeable alkaline or alkaline earth cations. These structural characteristics give them various properties such as ion exchange capability, solid acidity, adsorption, and catalytic capabilities, a fact that has allowed a large number of industrial uses to be discovered and initially promoted before the synthesis of new mineral species as well as cost optimization strategies for these syntheses. Referring to the latter, the optimization of processes are mainly channeled into shorter, simpler, and less energy-consuming times and processes, and the use and enhancement of waste or by-products from other industrial processes.

The zeolite GIS class has the typical oxide formula: M_2/n_O·Al_2_O_3_·1.80–5.00SiO_2_·5H_2_O where M is a n-valent cation, normally an alkali metal [1]. The sodium P zeolites are classified as synthetic counterparts of Gismondine-type (GIS) and their smaller micropore size (~2.9 Å) makes them useful and valuable applicants for the water vapor adsorption and separation of small molecules [2]. Albert et al. [3] indicate these zeolites as the most open framework-type generated thus far owning to the high flexibility of the Si-Al linkage.

Four different polymorphs have been recognized due to variable Si/Al ratio: the cubic phase called Na-P1, refined in *I4_1_*/*AMD* [4]; the orthorhombic phase—the so-called Na-P2 crystallizing in *Pnma* [5]; the tetragonal phase for high silica variety of Na-P refined in *I4_1_*/*AMD* [6]; and the monoclinic phase for P with a strict Si/Al ratio of 1.0 [3].

P zeolites are successfully used in water-softening [7], as components of environmentally friendly detergents [8], as sorbents of heavy metals [9], in gas separation applications [6], for the removal of radioactive waste species [1,8,10,11], and as stable support to prevent agglomeration in dispersing of particles in aldol condensation of cyclohexanone with benzaldehyde [12,13].

The standard procedure from the International Zeolite Association Synthesis Commission (IZA) is the hydrothermal method which indicates long crystallization times of 60 days [14].

Zeolite P has been synthesized in the past not only using the hydrothermal method [1,3,5,8,15,16,17], but also the microwave technique [18,19], the sol–gel process [20] and the sonochemical method [21].

The synthesis from low-cost silica-alumina sources has also been explored involving the use of fly ash [22,23,24,25], kaolinite [26], clays [20,27], nuclear wastes [28], waste glasses [29], transformation of natural mordenite and clinoptilolite [30,31,32], and rice husk silica [33,34,35,36,37,38].

With regard to the use of rice husk ash (RHA), Khabuanchalad et al. [33] reported the partial transformation of Y zeolite, synthesized from RHA, in NaP after a 5-day crystallization time; also, Wittayakun et al. [34] synthesized NaY from RH at 90 °C obtaining NaP as an additional product. Mohamed et al. [35] used RH to synthesize NaY obtaining a mixed intermediate product of NaY and NaP after mixing the seed gel and the feedstock gel at 110 °C for 24 h. Kongkachuichay and Lohsoontorn [36] used perlite and RH to synthesize analcime, Na-P1, and sodalite; RH was burned at 700 °C and the experiments were carried out in an autoclave with SiO_2_/Al_2_O_3_ molar ratios of 1 to 40, NaOH concentrations of 1 to 4 N, and starting pressure of 1 atm; the existence field of Na-P1 as the isolated phase is related to NaOH concentrations below 1.5 and to SiO_2_/Al_2_O_3_ molar ration between 5 and 9 at 140 °C. However, it must be said that an X-ray pattern, which testifies to the sole presence of the phase as a synthesis product, is neither provided nor other types of spectroscopic characterizations performed for the NaP zeolite. Furthermore, a cumulative characterization of the specific surface area and pore size is carried out without distinguishing between analcime, NaP, and sodalite. As far as the morphology of crystals is concerned, the authors generically indicate NaP as aggregates of various shapes. Referring to this point it should be said that Huo et al. [8] also reported the synthesis of NaP with different morphologies/crystalline habits. These authors synthesized NaP starting from chemical reagents and conducted a study aimed to define the influence of the SiO_2_/Al_2_O_3_ ratio in determining the shape of crystals.

The scope of the present work is to test RHA in the synthesis of high-purity monomineralic powders of Na-P1 (i.e., producing Na-P1 crystals characterized by a distinct morphology and homogeneous crystal size), working on the reduction in both the calcination and synthesis temperatures and the synthesis times and improving the degree of crystallinity of the synthesized powders (i.e., in terms of absence of amorphous phase and/or impurities coming from the raw material). Kongkachuichay and Lohsoontorn [36] state that zeolites are accompanied with trace amounts of unreacted perlite; at the same time, they define the synthesized zeolite as “pure” simply by comparing the peak positions to those of a collection of simulated XRD powder patterns of zeolites [39]. Furthermore, the work includes a quantitative phase analysis (combining Rietveld and reference intensity ratio methods) and a morphological, physical, and spectroscopic characterization for the Na-P1 zeolite obtained from RHA is provided here for the first time.

## 2. Results and Discussion

**Figure 1:**
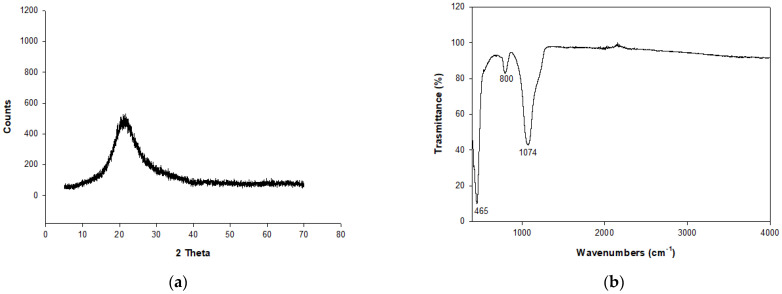
(**a**) X-ray spectra of RHA. (**b**) IR analysis on RHA.

The result of Powder X-Ray Diffraction analysis (PXRD) performed on RHA (Figure 1) reveals the amorphous character of the material (Figure 1a); a bulge around 20° 2 theta is in fact evidenced and indicative of non-crystalline material.

Infrared (IR) analysis was conducted on RHA (Figure 1b). It results in a strong band at 1074 cm^−1^ related to the asymmetric stretching vibrations of tetrahedral SiO_4_. A second minor band is located at 800 cm^−1^ associated with the symmetric stretching of SiO_4_ tetrahedra. The third strong and narrow band at 465 cm^−1^ is due to Si-O bending. The results agree with Petkowicz et al. [40], Yusof et al. [41], and Novembre et al. [42].

The percentage of the chemical composition of RHA determined by XRF is reported in Table 1, indicating a silica content of 98.55% and minor amounts of other oxides.

**Figure 2:**
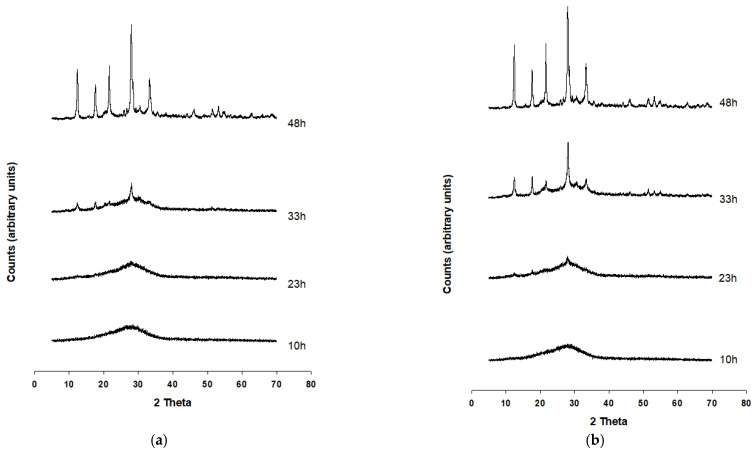
PXRD patterns of the synthesis run A (**a**) and B (**b**).

The synthesis runs conducted at 110 °C for experiments A and B are illustrated in Figure 2a,b. The appearance of Na-P1 zeolite is evident at 33 h for both the experiments. The reflection intensities increase reaching their maxima at 48 h. Comparing the two synthesis runs, it can be noted that the zeolitic crystallization seems to proceed at a greater speed in experiment B, as shown by the intensities of the counts at 33 h. It should be noted, however, that the intensities of the counts at the end of the runs appear comparable.

On the other hand, substantial differences in the morphologies of the crystals are highlighted through SEM analysis.

**Figure 3:**
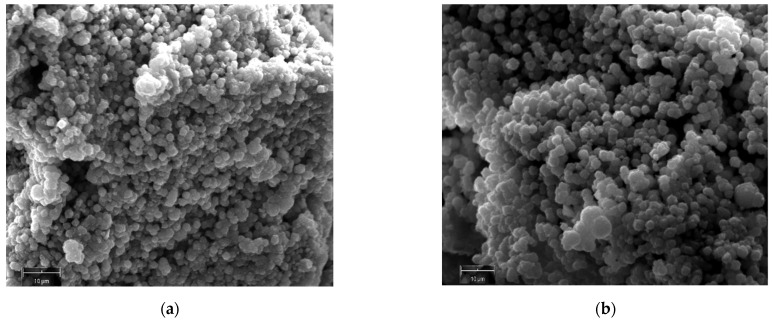
SEM images of Na-P1 zeolite crystals obtained at 48 h (110 °C) for experiment A (**a**,**b**).

Figure 3a,b present SEM images of Na-P1 crystals from experiment A at 33 h and 48 h, respectively. It reveals a microsphere morphology of the crystals and an average maximum size of crystals around 2 microns.

**Figure 4:**
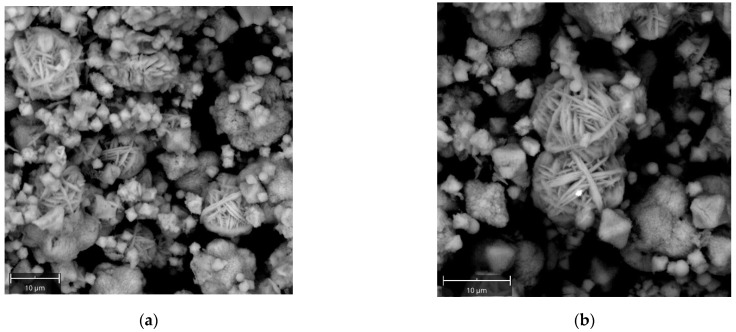
SEM images of Na-P1 zeolite crystals obtained at 48 h (110 °C) for experiment B (**a**,**b**).

Figure 4a,b report SEM images of Na-P1 crystals from experiment B at 48 h. In this case, different sizes and morphologies emerge for Na-P1 crystals. Besides crystals characterized by a microsphere morphology (5–7 microns), there are also wool ball-like-shaped crystals (around 10 microns). Together with these two morphologies another one is visible, associated with smaller crystals of about 1–2 microns characterized by an angular, pseudo-octahedral morphology.

Huo et al. [8] reported the synthesis of zeolite NaP with controllable morphologies starting from commercial reagents, i.e., sodium hydroxide, sodium aluminate, and sodium silicate. The authors explain how the SiO_2_/Al_2_O_3_ ratio plays an important role in morphology control. In particular, a mixture of different morphologies emerges for SiO_2_/Al_2_O_3_ ratios between 5 and 10. Also, in our case, a microsphere morphology is observed for SiO_2_/Al_2_O_3_ ratio of 3.5, while a coexistence of different morphologies is visible at the higher ratio of 5.3.

Given that the industrial processes require homogeneous products to perform chemical reactions with selected-sized molecules, we focused our attention on experiment A, which showed synthetic powders characterized by the presence of Na-P1 zeolite crystals of the same morphology and of dimensional homogeneity.

Further characterizations were therefore conducted on the sample produced at 48 h of experiment A.

The results of the QPA analyses are illustrated in Table 2.

**Figure 5:**
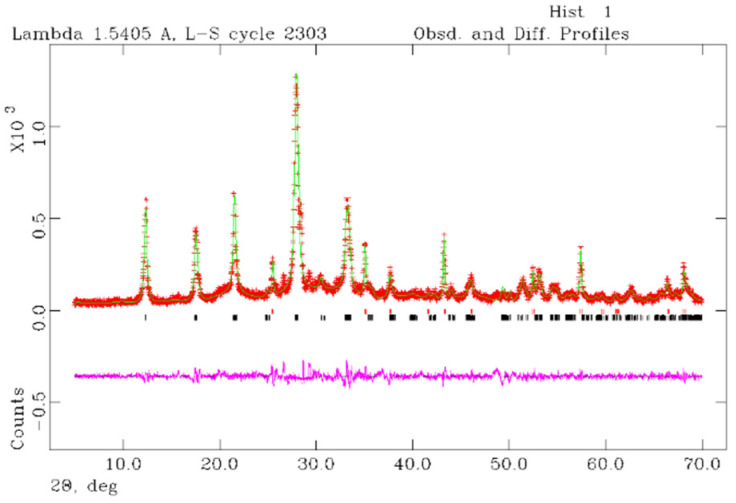
Rietveld refinement plot: observed (red +), calculated profiles (green) and difference plot (pink) for Na-P1 zeolite (110 °C-48 h) and corundum NIST 676a with tick marks at the position of the Bragg peaks. From the bottom: Na-P1 zeolite, corundum NIST 676a.

The observed and calculated profiles and difference plots for Na-P1 and corundum NIST 676a are reported in Figure 5. In particular, at 48 h, 93.5% of zeolitic phase is achieved. Cell parameters of Na-P1, refined with monoclinic symmetry space group *C*2/*c*, are reported in Table 2. The results of the Rietveld refinements provide cell values that are in good agreement with the structural model proposed by Albert et al. [3].

**Figure 6:**
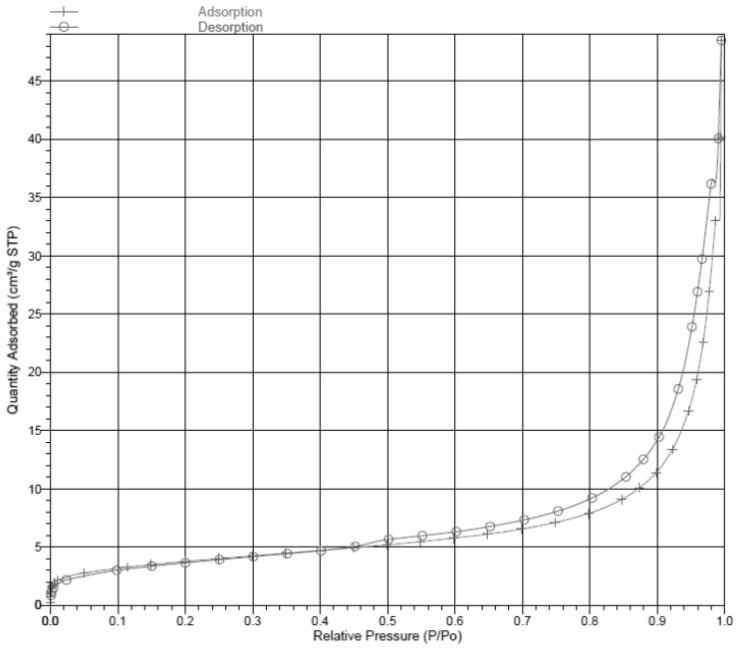
Nitrogen adsorption–desorption isotherms of the Na-P1 zeolite from experiment A.

Using the BET method, the obtained zeolites had a specific surface area of 13.27 m^2^/g and the average pore volume of 0.0071 cm^3^g^−1^. The specific surface area is rather small due to the low particle size of the sample, as confirmed by SEM analysis, and is also attributable to the fact that Na-P zeolite only has 8-membered rings in its structure, as previously observed by Breck, Huo, et al. and Ali et al. [8,14,43]. Figure 6 illustrates the nitrogen adsorption–desorption isotherms of the Na-P1 zeolite from experiment A. It results in a vertical hysteresis loop proving the existence of cylindrical mesopores in the zeolitic structure.

**Figure 7:**
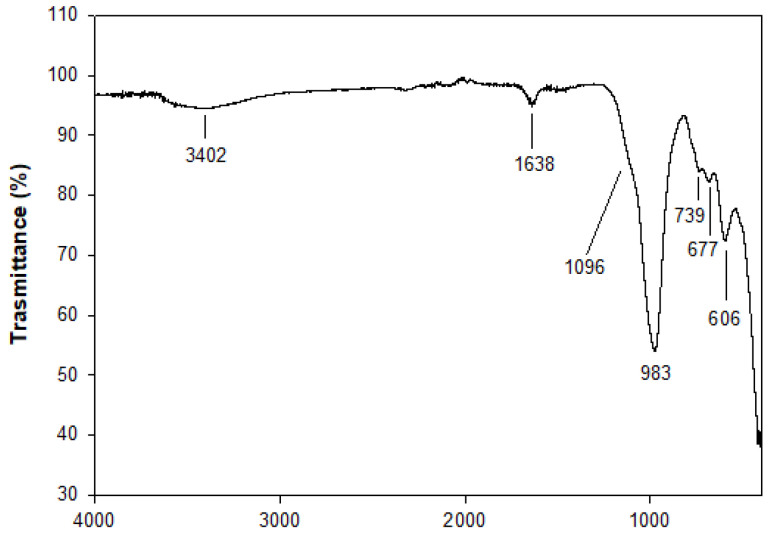
IR spectrum of the Na-P1 (48 h at 110 °C).

Figure 7 illustrates the infrared spectrum. The significant broad peaks are located at 3402 and 1638 cm^−1^ for sample 6A-48 h-110 °C; these peaks are related to O-H stretching and bending, respectively. The band at 1096 cm^−1^ is assigned to the asymmetric stretching vibration due to external linkages between tetrahedra, structure sensitive, sensu Flaningen et al. [44], and that at 983 cm^−1^ is assigned to the asymmetric stretching vibration caused by internal vibrations of the framework SiO_4_. The bands at 739 and 677 cm^−1^ are attributed to Si-O-Si symmetric stretching vibration of internal tetrahedron. The peak at 606 cm^−1^ is attributed to the double rings’ vibration. Data are coherent with those available in the literature [8,14,44,45].

**Figure 8:**
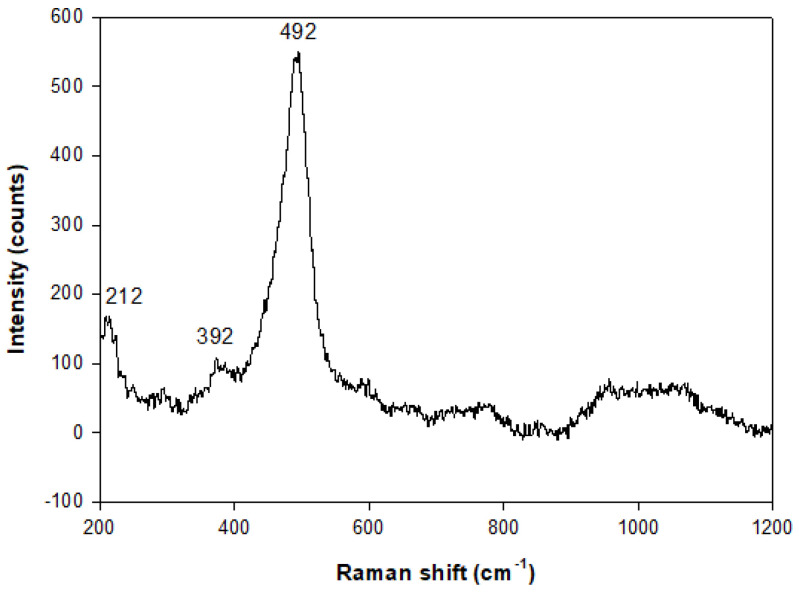
Raman spectrum of the sample at 48 h (110 °C) of experiment A.

Figure 8 reports the Raman spectrum for the sample 6A-48 h-110 °C. It results in a peak at 392 cm^−1^ related to the vibration mode of Al-O-Al and a peak at 492 cm^−1^ associated with the vibration mode of Si-O-Si. These data are coherent with findings by Tsai et al. [46] that report the characteristic peaks in the region of 391–432 cm^−1^ and 463–497 cm^−1^ for the doubly connected four-ring chain of the Gismondine group. Also, Mozgawa [47] indicates the bands in the range of 470–520 cm^−1^ due to the “breathing” vibrations of the 4-membered rings. The peak at 212 cm^−1^ is assignable to the vibration mode of Na-O.

**Figure 9:**
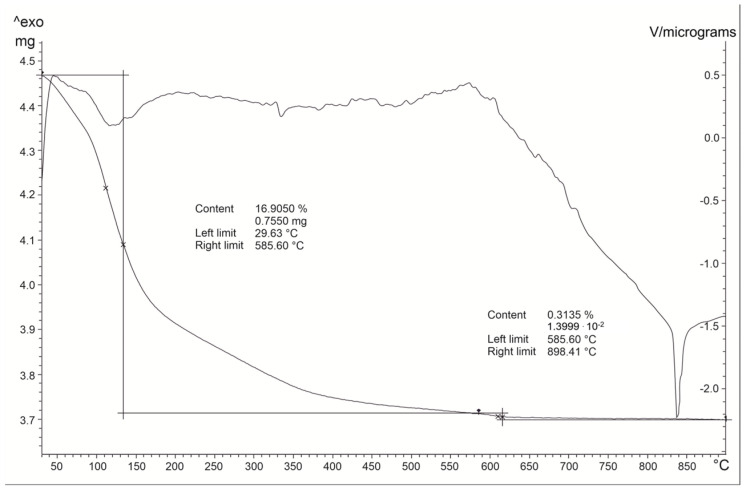
DTA-TG analysis of the sample at 48 h (110 °C) of experiment A. The asymptotic line to the x scale represents the TG analysis while the above line is related to DTA analysis.

Thermogravimetric analysis has revealed a gradual and continuous water loss up to 1000 °C (Figure 9). In particular, it indicates a two-stage mass loss. Physisorbed water is lost up to 120 °C. There also seems to be at least one more stage of water release between 110 and 150 °C which might be interpreted as evidence for the existence of two types of water molecules in the structure of different bound energies. C.a. 17% loss occurs at about 585 °C; between 585 °C and 900 °C a mass loss of 0.31% is registered and related to the removal of the crystal water. The endothermic peaks revealed by the DTA curve at 120 °C reflect the dehydration process and are in agreement with findings by Zubowa et al. and Huo et al. [8,18]. At about 838 °C there is an endothermic peak which testifies the melting of the phase.

## 3. Materials and Methods

Rice husk (RH) coming from a rice field on the northeastern coast of Spain underwent preliminary treatments: grinding, washing, and drying at 105 °C for 24 h. Then, calcination of RH was performed in an open porcelain crucible which was heated in a BE43N BICASA furnace (Bernareggio, Italy) to the calcination temperature of 550 °C at a pressure of 1 atm for 6 h; the heating rate of the sample was 1.5 °C s^−1^ [48,49]. Following the calcination treatment, RH ash (RHA) was obtained.

RHA composition was determined by X-ray fluorescence (XRF), with a WDXRF, Panalytical (Malvern, UK), Axios PW 4400/40 sequential spectrophotometer at the Centres Cientifics i Tecnològics de la Universitat de Barcelona (CCiT-UB). Major element determination has been carried out using fused pearls (lithium tetra borate pearls at a dilution of 1/20) according to the procedure by Novembre et al. [42,50], Gisbert and Gimeno [51], and Aulinas et al. [50]. In order to establish the XRF calibration curve, it is necessary to have a range of standards with a chemical composition similar to that of the unknown. Nevertheless, it is unusual to analyze materials with such a high silica content as RHA (and consequently it is not common to have standards for that composition). Therefore, an ad hoc procedure was developed to analyze ash with such a high silica content, i.e., mixing carefully 1:2 and 1:3 proportions of basaltic composition with RHA at international standards [52]; thus the final raw data resulted in the calibration range of the WDXRF for rhyolite-andesite. In the same way, taking into account the limitation of XRF methods in the calculation of Na in low contents, this fact was surpassed with an in-house laboratory calibration of raw data provided by WDXRF with inner standards and matrices of comparable composition, previously analyzed by AAS [53,54,55].

NaOH and NaAlO_2_ were used in the synthesis protocol (Honeywell Riedel-de Haen, Bucharest, Romania, purity of 99%).

The seed gels were prepared by mixing the silicate solution with the aluminate one according to the following procedure: 1 g of RHA dissolved in 30 mL NaOH (8%) solution; 1.4 g of NaAlO_2_ dissolved in 30 mL NaOH (8%) solution. The as-so-prepared mixtures were homogenized for two hours with a magnetic stirrer. Then, the silicate solution was gradually mixed with aluminate solution in the ratio of 1:0.5 (experiment A) and 1:0.35 (experiment B). The systems were left undisturbed for 24 h at room temperature.

The feedstock gels were prepared in the same way as the seed gels but without aging. The seed gels were then mixed into the feedstock gels and put inside stainless-steel hydrothermal reactors and heated at 10 °C/min until the desired temperature (110 °C) and kept for different times. Synthesis products were filtered with distilled water and dried in an oven at 40 °C for 24 h. The initial mixtures for experiments A and B had the following composition, respectively: 3.5 SiO_2_–1.00 Al_2_O_3_–11 Na_2_O–534 H_2_O and 5.3 SiO_2_–1.00 Al_2_O_3_–14.4 Na_2_O–664 H_2_O.

Powder X-ray diffraction (XRPD) analyses were performed to characterize RHA and products of synthesis; the instrument was a RIGAKU “MiniFlex II” (Rigaku, Osaka, Japan) operating with Bragg–Brentano geometry (CuKa = 1.5405 Å, 30 kV, 15 mA, 5–70° 2 theta scanning interval, step size 0.02° 2 theta, data acquisition speed of 0.033°/s). The identification of Na-P and relative peak assignment was performed with reference to the JCPDS code: 71-0962. Both the crystalline and amorphous phases in the synthesis powders were estimated using quantitative phase analysis (QPA) applying the combined Rietveld and reference intensity ratio (RIR) methods [56]; corundum NIST 676a was added to each sample, amounting to 10% (according to the strategy proposed by Novembre et al.) [57] and the powder mixtures were homogenized by hand-grinding in a mortar. Data for the QPA refinement were collected in the angular range 5–70° 2 theta with steps of 0.02° and 10 s step^−1^, a divergence slit of 0.5° and a receiving slit of 0.1 mm.

Data were processed with the GSAS software 3.0 [58] and the graphical interface [59] starting with the structural models proposed by Albert et al. [3] for Na-P1. The following parameters were refined: background parameters, zero shift, cell parameters, and peak profiles [60,61,62].

Morphological analyses were obtained by means of scanning electron microscopy, Phenom XL SEM–EDX (ThermoFisher Scientific, Dartford, UK); powders were analyzed with the following operative conditions: high vacuum, accelerating voltage of 15 kV, and 2–15 μm beam diameter.

The specific surface and porosity were obtained by applying the BET (Brunauer–Emmett–Teller) method with N_2_ using a Micromeritics ASAP2010 instrument (Norcross, GA, USA) operating from 10 to 127 kPa [63].

The IR spectrum was obtained using a Shimadzu IRAffinity-1S FTIR spectrophotometer (Shimadzu Italia S.r.l., Milan, Italy) equipped with a sealed and desiccated interferometer, a DLATGS (Deuterated Triglycine Sulfate Doped with L-Alanine) detector, and a single reflection diamond ATR crystal (QATR 10, Shimadzu Italia S.r.l., Milan, Italy) [64]. The FTIR spectrum was recorded in the range of 4000–400 cm^−1^ co-adding 45 interferograms at a resolution of 4 cm^−1^ with Happ–Genzel apodization [64]. The ATR crystal was carefully cleaned before each analysis, a background was recorded for each sample and the measurements were performed in triplicate. Spectra manipulation was carried out with the software LabSolution IR version 2.27 (Shimadzu Italia S.r.l., Milan, Italy).

The Raman spectrum of NaP-1was obtained by confocal and high-performance Raman microscope XploRA PLUS (Horiba, Kyoto, Japan) with deep-cooled CCD detector technology. LabSpec 6.6.1.14 (Horiba, Kyoto, Japan) was employed to control, optimize, and process the acquired data. Furthermore, data were processed through Origin 8.5 to optimize the results. The analysis was performed in the range of 200–1200 cm^–1^ and with an 1800-line/mm grating.

Differential thermal analysis (DTA) and thermogravimetry (TG) were performed on the zeolitic powder using a Mettler TGA/SDTA851e instrument (Columbus, OH, USA) with the working conditions of 10°/min, 30–1100 °C, and a sample mass of ~10 mg in an Al_2_O_3_ crucible [65,66].

## 4. Conclusions

In this paper, we have successfully synthesized Na-P1 crystals starting from an environmentally friendly and inexpensive precursor constituted by RH. RH was pyrolyzed at 550 °C to obtain RHA. The experimental protocol of synthesis involved the mixing of a seed gel and a feedstock gel and crystallization at ambient pressure at 110 °C. Two different conditions were investigated by fixing the SiO_2_/Al_2_O_3_ ratio at 3.5 for experiment A and at 5.3 for experiment B. The appearance of the Na-P1 phase begins at about 24 h and the climax in the crystallization is reached at 48 h for both the experiments. Morphological study of synthetic powders reveals a ball-like shape and crystalline dimensional homogeneity (about 2 microns) for Na-P1 crystals of experiment A and different sizes (from 2 to 10 microns) and mixed morphologies (ball-like, angular and wool ball-like) for crystals of experiment B.

The complete spectrum of spectroscopic, physical, and morphological characterizations of zeolite Na-P1 synthesized starting from RHA at 48 h (experiment A) is presented here for the first time and shows that the products obtained are homogeneous in size and morphology, a factor mandatory in several industrial fields, i.e., gas separation. This demonstrates the effectiveness of the synthesis protocol by proving the validity of using RHA as a substitute for commercial silica.

Last but not least, we reached a reduction in the calcination temperature of RH and synthesis temperature. Among the authors that synthesized Na-P1 from RH [34,35,36], only Kongkachuichay and Lohsoontorn [36] managed to obtain the zeolite as single phase; they first pyrolyzed RH at 700 °C and then operated hydrothermal treatment at 140 °C. We have reduced calcination and hydrothermal temperatures to 550 °C and 110 °C, respectively.

Another substantial difference between our work and that of previous authors lies in the effective assessment of the degree of success of the experiment from calculation by the QPA of the percentage of crystallization vs. amorphous material and other impurities [67]. This is a fundamental requirement for an industry that claims at least 90% of pure products. Samples at 48 h of experiment A reached 93.5%.

To conclude, the comparison with the characterizations of the same zeolite obtained from traditional industrial chemical reagents suggests that transfer to an industrial production scale might be feasible.

## Figures and Tables

**Table 1:** Percentage of chemical composition of RHA determined by XRF.

SiO_2_	Al_2_O_3_	Fe_2_O_3_	CaO	Na_2_O	K_2_O	MnO	TiO_2_	MgO	P_2_O_5_
98.55	0.28	0.16	0.16	0.18	0.22	0.16	0.02	0.14	0.13

**Table 2:** Results of the QPA analyses conducted on samples synthesized at 170 °C.

Sample + 10% Corundum Nist 676a	110 °C-48 h
R_wp_	0.18
R_p_	0.15
CHI^2^	2.39
space group Na-P1	*C*2/*c*
*a* (Å)	14.3027 (0.0023)
*b* (Å)	10.0857 (0.0035)
*c* (Å)	10.0129 (0.0042)
*β* (°)	135.2833 (0.0031)
% amorphous	6.5 (15)
Na-P1	93.5 (18)

## Data Availability

The original contributions presented in this study are included in the article. Further inquiries can be directed to the corresponding author.
